# Parental–Caregivers Perceptions Questionnaire (P-CPQ): translation and evaluation of psychometric properties of the French version of the questionnaire

**DOI:** 10.1186/s12903-018-0670-8

**Published:** 2018-12-11

**Authors:** Noeline Razanamihaja, Marie-Laure Boy-Lefèvre, Laurence Jordan, Lea Tapiro, Ariane Berdal, Muriel de la Dure-Molla, Sylvie Azogui-Levy

**Affiliations:** Universite Paris Diderot, Paris, France

**Keywords:** Quality of life, Oral health, Children, Reliability, Validity

## Abstract

**Background:**

The Parental–Caregivers Perceptions Questionnaire (P-CPQ) is a measure of parental/caregivers’ perceptions of the impact of children’s oral health on quality of life. The aim of the study was evaluate the psychometric properties of the French version of the P-CPQ.

**Method:**

The original P-CPQ was developed in English language and has 31 items divided into four sub-scales. This cross-sectional study used the translation-back translation method. The translated questionnaire was pretested on 14 parents-caregivers to obtain the final French version. The psychometric properties were tested on 142 parents/caregivers of three clinical groups of children from 8 to 10 years old without dental/facial anomalies (presumed healthy), with oral-facial clefts and with oral-dental anomalies linked to a rare disease other than cleft, approached in the waiting room of the Centre of the Hospital Rothschild in Paris, France, where the children attended treatment. Internal consistency was assessed by Cronbach’s alpha and test-retest reliability by Intra-class Correlation Coefficient (ICC). Construct validity was measured by correlations between the total scores and the global ratings of oral health and overall wellbeing, and tested using exploratory factor analysis (EFA) and the factorial structure was evaluated by the partial confirmatory factor analysis (PCFA). Discriminant validity was determined using Kruskall-Wallis test.

**Results:**

The mean (standard deviation) P-CPQ score was 18.73(18.79). Internal consistency was confirmed by a Cronbach alpha of 0.85. The test-retest reliability revealed that the responses to items were satisfactorily stable (ICC = 0.88). Construct validity was demonstrated by significant correlation coefficients between the total scale and the global ratings (r = 0.54 and 0.46; p < 0.001). Factor analysis with Principal Component Analysis extracted seven factors explaining 65.23% cumulative variance. Goodness-of-fit indices for partial confirmatory factor analysis were satisfactory for the 7-factors model of the French-PCPQ version. There were statistically significant differences between clinical groups regarding the total scale, thus demonstrating discriminant validity (p < 0.001).

**Conclusion:**

This French P-CPQ version showed reliability and validity comparable to the previous versions. However, the cross-cultural structure of the subscales should be further evaluated.

**Electronic supplementary material:**

The online version of this article (10.1186/s12903-018-0670-8) contains supplementary material, which is available to authorized users.

## Introduction

It is now well documented that oral diseases can affect both daily life and quality of life [1, 2]. Oral Health related Quality of Life (OHRQoL) is recognised by the World Health Organisation (WHO) as a component of general health that is now part of the global oral health programme [3]. Children are subject to numerous oral-dental anomalies and oral diseases and these could have a considerable impact on their daily quality of life and that of their family [4]. Thus, interest in oral health related quality of life has grown considerably over the past few decades [5]. Authors have reported that measuring oral health related quality of life could help in the evaluation of treatment needs, the prioritisation of care and the evaluation of treatment results [6]. Consequently, establishing the psychometric properties of an instrument capable of assessing it is essential prior to its administration in clinical practice and research.

One of the most frequently used instrument developed to this end is the Child Oral Health Quality of Life (COHQoL): a series of questionnaires developed in Canada by Jokovic et al. The COHQoL is composed of questionnaires for measuring: a) children’s perceptions, the CPQ _5–7_, the CPQ _8–10_ and CPQ _11–14_ (for children aged from 5 to 7; 8 to 10 and 11 to 14 years old respectively) [7, 8]; b) the perceptions of parents/caregivers, the Parental-Caregiver Perceptions Questionnaire (P-CPQ) [9]; and c) the impact of children’s oral health on family life: Family Impact Scale (FIS) [10]. The P-CPQ and FIS questionnaires are linked to the oral health of children to be filled in by the parents while the CPQ must be filled in by the children themselves according to their age group. Recently, two short forms of the P-CPQ have been developed with 16 items and 8 items respectively [11]. According to Malden et al., the P-CPQ and the FIS are evaluation measures that can be used in research on dental health care [12]. The P-CPQ originally developed in English for the USA, has been translated and cross-cultural adapted in Canada (in English) [9]. China [6], Brazil [13–15], Peru [16], the UK, [17], New Zealand [11] and USA [18]. These studies have all demonstrated good psychometric properties. Psychometric properties refer to the reliability and validity of the instrument. According to Guillemin et al., if the questionnaire is to be used in another country with a different language and culture, both translation and cultural adaptation will be necessary [19]. Given the parental influences on decisions making for their children health, the lack of a French version of the P-CPQ will limit its use for oral health research and use in paediatric dental services in France. Therefore, the objectives of this study were to translate and evaluate the reliability and validity of the French version of the P-CPQ.

## Material and methods

### The questionnaire

The P-CPQ began with two questions asking parents for a global rating of their children’s oral health: “How do you evaluate the health of your child’s teeth, lips, jaws and mouth?” (Choice of Responses: Excellent, Very good, Good, Fair, Poor) and “To what extent is your child’s well-being affected by the state of their teeth, lips, mouth and jaws?” (Possible responses: Not at all, Very little, Some, A lot, Very much). The P-CPQ is composed of 31 items divided into four sub-scales including: Oral Symptoms (OS) (6 items), Functional Limitations (FL) (7 items), Emotional Well-Being (EWB) (8 items) and 10 items for Social Well-Being (SWB). The questions referred to the frequency of events occurring during the previous 3 months. A-five point Likert-like scale was used with the following options of response: “Never” (score 0), “Once or twice” (1), “Sometimes” (2), “Often” (3) and “Nearly every day” (4).

### Study site

This cross-sectional study was performed in the framework of the Odontology Centre of the Hospital Rothschild in Paris (Assistance publique Hôpitaux de Paris), France.

### Study population and sampling

The psychometric properties were evaluated on a sample of 142 parents/caregivers of children between 8 to 10 years old. The study focused on parents accompanying their children. Inclusion criteria were: participants must be able to read, write and self-administer the questionnaire. Parents or caregivers coming from different locations in Paris and even from farer regions were approached with a consent form and the questionnaire through their children who underwent clinical examinations at the clinical centre. Children were divided into three groups before the beginning of the data collection according to their oral health conditions. The groups were:

1. a group of children attending paediatric odontology consultation and who presented no dental or oral/facial anomaly (healthy group)
2. a group of children with oral-facial clefts (clefts group),
3. a group of children presenting dental anomalies linked to a rare disease other than oral/facial clefts (structure, number and size anomalies) (others).

Parents were informed on the objectives of the study and had given their written consent to answering the questionnaire.

### Sampling

A convenience sample was selected for this cross-sectional study. Sample comprised parents/caregivers accompanying their children at the Odontology Centre of the Hospital Rothschild in Paris. To determine the minimum sample size required we referred to the Everitt et al.’s recommendation for a sample size ranging from 100 to 200 [20] and to a “ 4 subjects to 1 item ratio”. One hundred forty two parents/caregivers of children aged 8 (*n* = 52), 9 (*n* = 33) and 10 years old (*n* = 57) agreed to participate in the survey.

### Data collection and ethics

Two interviewers approached the parents by way of an informative letter, a consent form that they were asked to sign and the French P-CPQ questionnaire. Parents were invited by the two interviewers to self-complete the questionnaire in the waiting room of the odontology centre while their children underwent clinical examination.

The study was approved by the Research Project Ethics Evaluation Committee of the Hospital Robert Debré (AP-HP), under approval N° 2016/250–2.

The original version of the P-CPQ was obtained using an article available with open access, shared by the terms of “Creative Commons License” which allowed its use without restriction, distribution and reproduction, with the necessary condition of citing the authors’ works.

### Adaptation of the French version of the P-CPQ

**Fig. 1:**
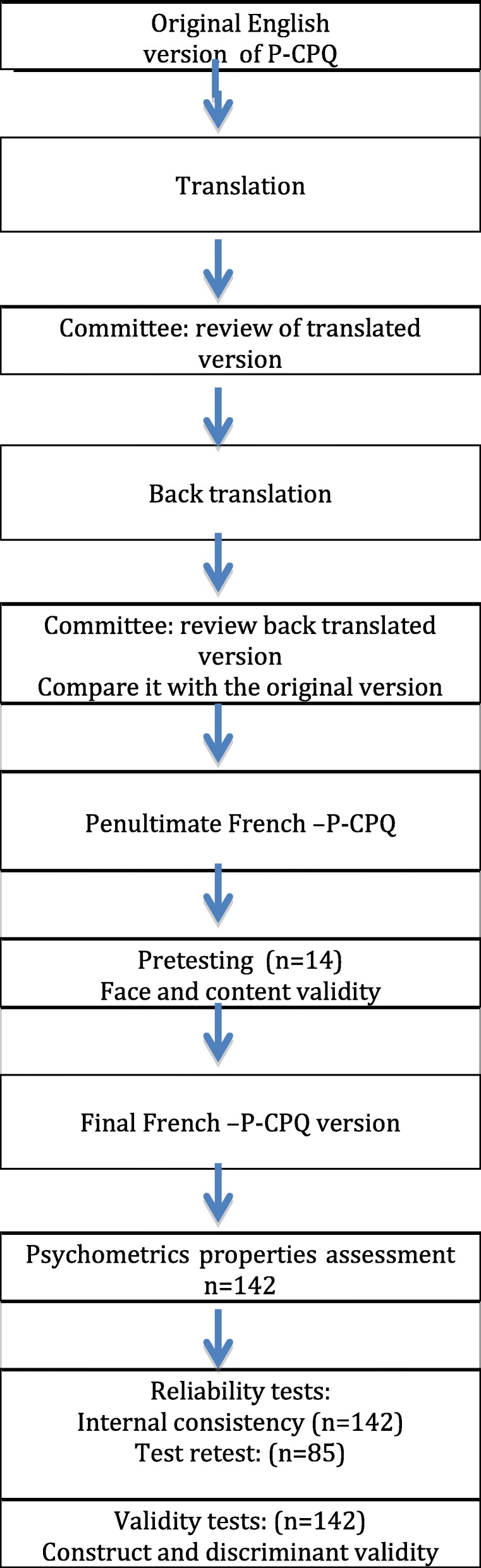
Flow chart of the translation steps and cross-cultural adaptation

The steps of the translation and the evaluation process of psychometric properties of the French-P-CPQ are presented in a flow chart. (Fig. 1) The sociocultural and conceptual adaptation of the P-CPQ was carried out according to the specific recommendations for the self-administered measures [19, 21].

### Forward–back translation

First, two independent translators translated the P-CPQ questionnaire from English to French. A committee met to decide on a single translation, which was then back translated into English by two other translators including a dentist and a linguist with perfect knowledge of English. Both were totally unaware of the existence of the original version. The committee of experts composed of all the translators and the researchers compared the two back-translations with the original in English, and then revised and agreed on the penultimate French version.

### Pre-test

The penultimate version was subjected to a pre-test on a group of fourteen parents/caregivers of children 8 to 10 years old who did not belong to the final sample, in order to discuss the pertinence of items and to identify problems of comprehensibility. The main purpose of the pre-test was to detect misunderstandings, ambiguities, or other problems participants may encounter with the questions and response options. The respondents were briefed about the purpose of the pre-test. They were invited to highlight questions they think difficult to understand. They were also asked to give comments on the clarty of the questionnaire. This led to the revision of the questionnaire by the committee and the final version of the French-P-CPQ was obtained. (Additional file 1).

### Statistical analysis

The P-CPQ scores for each participant was calculated by summing the valid responses scores to all items, whereas the domain’ scores were obtained by summing the items scores within each of the four domains. As there were 31 items, the final score could vary from 0 (no impact on the children’s Oral Health Quality of Life) to 124 (maximum impact). The highest scores indicated the highest levels of negative impact of oral conditions on the child’s quality of life perceived by the parents. “I don’t know” was permitted as response to an item and was given a score of 0(zero) as a “never” option. The choice of zero was based on data from Jokovic et al. [9].

The SPSS 24.0 software for Windows was used to process and analyse the data. Initially, demographic data was presented in numbers and percentages. Descriptive analysis were also performed to assess the frequency distribution of oral impacts and measures of central tendency (means and standard deviation) of the total scale and subscales scores of the French-P-CPQ. Ceiling or floor effects was evaluated on the basis of the percentage of respondents with the maximum or minimum P-CPQ score and was considered present if this was in 15% or more of the respondents [22]. The second step of the analysis included tests to confirm the reliability and validity of the French version of the P-CPQ. Reliability was assessed using two methods: test-retest reliability and internal consistency reliability. The reliability of the French version of the P-CPQ was tested on the parents (*n* = 85) who, two weeks later, filled the questionnaire once again for the retest. Test-retest reliability was evaluated using Intra-Class Correlation (ICC) calculated by two-way analysis of variance [23]. Reliability was considered acceptable if the ICC was higher than 0.7 and if the 95% confident interval of an ICC estimate is 0.83–0.94, the level of reliability can be regarded as “good” to “excellent” [24]. Internal consistency was evaluated using Cronbach’s alpha with values α > 0.7 considered acceptable [25].

### Construct validity

To analyse construct validity, the associations between French-P-CPQ scores and the two global indicators were determined using Spearman’s rho correlation coefficient. The two questions presented separately at the beginning of the text were considered for the construct validity and Spearman rho correlation was used for this purpose. Therefore, associations were analysed between total scale score and subscales scores with the global ratings of oral health and overall wellbeing. The hypothesis tested was that parents reporting negative impact of children’s oral health on their every day life and poor oral health status have higher P-CPQ scores than parents reporting positive impact or good oral health. Factor analysis was used to evaluate the structure of the French-P-CPQ version to confirm whether the questionnaire contains appropriate groups of constructs and items. In the present study, the Exploratory Factor Analysis (EFA) with the extraction of factors by component principal analysis (CPA) and varimax rotation with Kaiser normalisation was carried out to evaluate the factor structure of the French-P-CPQ scale. Before performing factor analysis, Kaiser-Meyer-Olkin (KMO) criteria factors and Bartlett’s sphericity test were assessed to evaluate the justifiability of performing factor analysis on the French-P-CPQ. The KMO value of ≥0.80 indicated sample size accuracy and justifiability of factor analysis. The eigenvalue> 1 and scree test, were used to determine how many factors to retain. Kaiser’s criterion suggests retaining all factors that are above the eigenvalue of 1. A KMO > 0.6 and variance explained by loaded factors> 60% were considered. A variable loads strongly if its loading is ≥.40 in a particular factor and is considering clean if its absolute difference between loadings exceeds 0.20 [26].

EFA was followed by a partial confirmatory factor analysis (PCFA) to verify the goodness of fit of the model that was built according to the results of EFA. PCFA was performed to validate the factorial validity of the model derived from the results of the EFA. For this, we have performed PCFA with Maximum Likelihood Estimation method, and by calculating the implied model chi-square (Goodness-of-fit test) and the degree of freedom associated with the residual correlation matrix. The residual correlation resulted was used in SPSS to estimate the value of the Standardized Roots Mean Residual (SRMR) value that would get from a CFA [27]. Thus, with SPSS, to calculate an incremental close fit index, two chi-square values were required (the chi-square obtained by Barlett’s test of sphericity and the implied chi-square) with the respective degree of freedom. Model fit verification was done using normed chi square test and several different indices of fit including the Normed Fit Index (NFI), Comparative Fit Index (CFI), Tucker Lewis Index (TLI) and Root Mean Squared Error for Approximation (RMSEA). A normed chi^2^ < 3 is considered a good model. An acceptable scores of CFI, NFI was >.90 respectively; TLI = .90 and RMSEA value of 0.06 and lower was indicative of a good model fit [28]. A cut-off value of .05 was used for SRMR and RMSEA. Cronbach’s α was also determined for reliability of the extracted 7-factors.

### Discriminant validity

For discriminant validity assessment, three groups were compared; parents of children presumed healthy without dental/facial anomaly (group 1), group of children of oral/facial cleft (group 2) and third group of children subject to oral/dental anomaly linked to a rare disease other than cleft (group 3). To test the discriminant validity, the hypothesis was that scores would be higher among parents/caregivers with children in cleft group (group 2) and the oral/dental rare diseases, other than cleft (group 3), and lower in presumed healthy group (group 1). A Shapiro-Wilk test of normal distribution was performed and a non normal distribution of the results was observed. Because the test was significant (*p* < 0.05) thus the null hypothesis was rejected which confirmed that the data were not normally distributed. Since the P-CPQ scores were not normally distributed, the Kuskall-Wallis nonparametric test was used to determine whether the parents of children with oral/palatal cleft and/or linked to rare disease perceived more impact on their children’s quality of life than those children were assumed to be healthy. The level of significance was set at 0.05. Criterion validity was not applicable for this study as there is no gold standard.

## Results

### Translation

The expression “sores in mouth” in English, which means “*plaie dans la bouche*” in French, seemed ambiguous and was finally translated into “*douleurs dans la bouche*” (pain in mouth). Since chewing “corn on the cob” is not suitable for the nutritional habits of children in France, it was excluded. The pre-test results showed that the French-P-CPQ was well understood by the respondents.

### Demographic characteristics

The mean (standard deviation) age of children was 9.04(0.88), of which 74(52.1%) were boys and 68(47.9%) girls. A total of 142 parents of children aged from 8 to10 years old answered the P-CPQ questionnaire among whom 84 (59.1%), 23 (16.2%) and 35 (24.6%) were respectively parents of healthy children (with no oral-facial malformation) = *health group*, parents of children with oral-facial clefts = *cleft group* and parents of children with anomalies linked to a rare disease other than clefts = *other group*. 105(73.3%) of the respondents were mothers, 26(18.3%) fathers and 11(7.7%) were others caregivers. (Table 1).

**Table 1:** Characteristics of parents/caregivers and children

Characteristics	n	%
Parents/caregivers by Children age		
8 years old	52	36,6
9 years old	33	23,2
10 years old	57	40,1
Children gender		
Male	74	52.1
Female	68	47.9
P-CPQ completed by		
Child’s mother	105	73,3
Child’ father	26	18.3
Others	11	7.7
Clinical groups		
Healthy group	84	59,15
Clefts group	23	16,20
Oral rare diseases Other than clefts	35	24,65
Total	142	100,0

The number of “I don’t know” (DK) responses per parent was very limited. Only three parents had ticked DK as responses to one item of emotional wellbeing or social wellbeing. Overall, 94.4% of the parents reported oral impact. In the study population, the scores for the total scale from 0 to 81 with a mean (standard deviation) of 18.73(18.79), 90.8% of them reported experiencing Oral Symptoms in the previous three months, 75.4% reported Functional Limitations, 72.5% reported Emotional Well-Being, and 64.1% reported Social Well-Being. The frequency of a total score of zero was 5.6% not exceeding 15% (no floor effect). No respondent achieved the maximum possible score (no ceiling effect) of the P-CPQ. Thus, no floor or ceiling effects in total scores. In subscales, FL, EWB and SWB, > 15% of respondents scored “never” (Table 2). The proportion of individuals who achieve the lowest numeric value of a score was found in the “healthy” group.

**Table 2:** Descriptive statistics for the French-P-CPQ total scale and subscales scores and sample distribution according to floor and ceiling effects

			Range	Floor effect		Ceiling effect	
	Mean (SD)	%		n	%	n	%
Total score (0; 132)	18.73 (18.79)	94.4	0; 81	8	5.6	0	0.0
Subscales							
OS (0; 28)	5. 27 (3.96)	90.8	0; 20	13	9.2	0	0.0
FL (0; 32)	5. 72 (6.36)	75.4	0; 30	35	24.6	0	0.0
EWB (0; 32)	4. 01 (4.81)	72.5	0; 26	39	27.5	0	0.0
SWB (0; 40)	3. 72 (5.45)	64.1	0; 26	51	35.9	0	0.0

*SD* standard deviation

### Internal consistency and test-retest reliability

Cronbach’s alpha of the total scale score was 0.85, indicating excellent internal consistency and for the subscales, values ranged from 0.77 for Social wellbeing to 0.81 for Oral Symptoms. The reliability of the French-P-CPQ according to the test-retest measured by the intra-class correlation coefficient (ICC) provided a global value of 0.88, CI 95% [0.82–0.92]. ICC values varies from 0.76 (Functional Limitations) to 0.86 (Social Wellbeing) (Table 3).

**Table 3:** Internal consistency reliability and test retest reliability statistics

P-CPQ	Number of Items	Cronbach’s alpha (*n* = 142)	Intraclass Correlation Coefficient (*n* = 85)	
			ICC	95% CI
Total scale	33	0.85	0.88	[0.82–0.92]
Subscales				
Oral Symptom (OS)	6	0.81	0.81	[0.71–0.88]
Functional Limitation (FL)	8	0.77	0.76	[0.64–0.84]
Emotional Well-Being (EWB)	7	0.79	0.79	[0.68–0.86]
Social Well-Being (SWB)	10	0.77	0.86	[0.79–0.91]

*ICC* intraclass correlation

### Construct validity

The correlations between global ratings (Oral health and overall wellbeing) and the total score (*r* = 0.54 and *r* = 0.46), the subscales: oral symptoms (*r* = 0.41 and *r* = 0.38), functional limitations (*r* = 0.38 and *r* = 0.34), emotional wellbeing (*r* = 43 and *r* = 43), social wellbeing (*r* = 45 and *r* = 41) were weak but highly significant (*p* < 0.001) (Table 4).

**Table 4:** Construct validity: spearman rho correlation coefficients between total scale scores and global ratings of oral health and overall wellbeing

Variables	Global ratings			
	How would you rate the health of your child’s teeth, lips, jaws and mouth? [Oral health]		How much is your child’s overall wellbeing affected by the condition of his/her teeth, lips, jaws or mouth? [Overall wellbeing]	
	*r* ^a^	*p*-value	*r* ^a^	p-value
Total Scale	0.54	< 0,001	0.46	< 0,001
Subscales				
OS	0.41	< 0,001	0.38	< 0,001
FL	0.38	< 0,001	0.34	< 0,001
EWB	0.43	< 0,001	0.43	< 0,001
SWB	0.45	< 0,001	0.41	< 0,001

^a^Spearman’s rank correlation coefficien*t*

We carried out an exploratory factor analysis EFA on the answers of the respondents to the 31 items. Before EFA, the Kaiser-Meyer-Olkin (KMO) test and Bartlett’s test of sphericity were conducted to evaluate the factorability and ensure that EFA was adequate for principal component analysis (PCA). The Kaiser-Meyer-Olkin test indicated good adjustment to latent factors (KMO = 0.885) and Barlett’s test of sphericity was significant at *p* < 0.001 meaning that EFA can be applied to the dataset. An exploratory factor analysis was conducted to determine the factor structure of the French-P-CPQ and to explore item placement within factors. Varimax rotation was used. This procedure led to a different grouping from the initial framework and the 31 items of the French-P-CPQ were loaded into 7 different factors with Eigenvalues> 1 and the total variance explained was 65.23%. The first factor taken alone explains 36.33% of the total variance of the 31 items in the analysis which included: 1-First factor: consists of the totality of the original emotional wellbeing subscale (7/7 items) [14–20], and social wellbeing subscale (5/10 items)(items: 2, 25, 27, 28, 29) related to “relationship”; 2-Second Factor. “Eating difficulties”: represents 4/8 items of functional limitations domain (items 7, 11, 13, 14); 3-Third factor: “oral dental/pain” (items 1, 3, 4, 12); 4-Forth factor: “Oral Symptom and discomfort ”(items 5, 9, 24, 26); 5-Fith factor: “functional limitation” (items 8, 10); 6- Sixth factor: “discomfort”: (items 22, 30, 31) and 7-Seventh factor: “oral hygiene” (items 2, 6).. (Table 5) The maximum likelihood estimation analysis values associated with the PCFA solution was equal to 391.315 with 269 degree of freedom (df) and *p* < 0.001 which is smaller than the corresponding null model chi square Bartlett’s test of sphericity of 2443.025 and *p* < 0.001. Using the Kaiser criterion of retaining all components with eigenvalues above 1, 12 items loaded strongly (0.4+) on the first component; 4 items loaded strongly (0.4+), or on the second, third and fourth component respectively; 2, 3 and 2 items loaded strongly (0.4+), on the fifth, sixth and seventh component respectively. Item 13 (difficulty eating foods he/she would to eat) was identified to have the highest factor loadings of 0.856, while item 23 (hard time paying attention in school) had the lowest factor loadings of 0.443. The reliability of the 7-factors model was tested and the results showed a scale Cronbach’s alpha of> 0.80.

**Table 5:** Exploratory factor analyses of the P-CPQ: factor loadings from the structure matrix

Item wording	Components						
	Factor1	Factor2	Factor3	Factor4	Factor5	Factor6	Factor7
	Relationship	Eating difficulties	Oral/dental pain	Oral Symptom and discomfort	FL	Social Discomfort	Oral hygiene
SOS (6 items)							
1. Pain in teeth			.703				
2. Bleeding gums							.719
3. Sores in mouth			.694				
4. Bad breath			.544				
5. Food stuck to roof of mouth				.796			
6. Food caught							.483
FL (8 items)							
7. Difficulty biting, chewing firm foods		.630					
8. Breathing through mouth					.768		
9. Trouble sleeping				.460			
10. Unclear speech /					.605		
11. Slow eating		.587					
12. Difficulty drinking/eating hot/cold foods			.496				
13. Difficulty eating foods would like to eat		.856					
14. Breathing through mouth					.768		
13. Restricted diet		.790					
EWB (7 items)							
15. Upset	.480						
16. Irritable/frustrated	.637						
17. Anxious/fearful	.567						
18. Worried that is different from other people	.742						
19. Worried he/she is less attractive than other	.795						
20. Shy/embarrassed	.741						
21. Worried having fewer friends	,642						
SWB (10 items)							
22. Missed school						.502	
23. Hard time paying attention in school	.443						
24. Not wanted to speak/read aloud in class				.524			
25. Not wanted to talk to other children	.663						
26.Avoided smiling when around other children				.501			
27. Teased/called name by other children	.667						
28. Left out by other children	.680						
29. Not wanted/unable to be with other children	.753						
30. Not wanted/unable to take part in activities (sport, … clubs)						.712	
31. Asked by other children about condition						.518	
% of variance (Total variance explained = 65.23)	36.33	7.79	5.18	4.64	4.34	3.55	3.40

Extraction method: *PCA* principal component analysis

Rotation method: Varimax with Kaiser normalisation

Based on khi^2^ values and their degrees of freedom, the close fit indices were calculated to be NFI = 0.837; CFI = 0.939; TLI = 0.889; RMSEA = 0.049 and SRMR = 0.036 (Table 6).

**Table 6:** Quality of goodness-of-fit indices

		Threshold of acceptability	
Indices	Model	Good fit	Acceptable fit
Khi^2^	391.315	0 ≤ Khi^2^ ≤ 2df	2df ≤ Khi^2^ ≤ 3df
df	269		
p	.000		
Khi^2^/df	1.45		Khi^2^/df < 3
NFI	.837	NFI > .90	
CFI	.939	CFI > .90	
TLI	.889	TLI > .90	
RMSEA	.049	0 ≤ RMSEA≤.06	.06 ≤ RMSEA≤.08

The Visual Summaries of Fit of distributions of the residuals showed that the frequency distributions of the correlation residuals or covariance residuals had a normal shape.

When only the first 4-factors, were retained, (eigenvalue> 1), these explained 47, 241% of total variance. Factor 1: contained 15 items from EWB (5/7items) and SWB (9/10 items); Factor 2: were found 7/8items of FL; Factor 3: was composed by 6 items: 2 items moved from EWB ( “been upset” and “irritable and frustrated” and 3 items moved form SO (bleeding gum; bad breath and food caught); and 1 item from SWB “Not wanted/unable to take part in activities (sport, drama, clubs)” . Factor 4 with only one item can’t be considered as a factor. It’s about “Food stuck to roof of mouth” . The values of absolute fit indices showed a poor level of fit: khi^2^/df = 1.69; NFI = 0.759; TLI = 0.837; CFI = 0.879; RMSEA = 0.073.

*Discriminant validity* confirmed the hypothesis. As expected, the mean of total scale score was higher for parents/caregivers with children in the cleft and other rare disease groups and lower in the paediatric healthy group. The difference between groups reached statistical significance (*p* < 0.001 and *p* < 0.05) (Table 7).

**Table 7:** Discriminant validity: overall and subscale scores for healthy group, clefts group and Oral rare diseases other than clefts group

Variables	Healthy group (*n* = 84)			Clefts (*n* = 23)			Oral rare diseases other than clefts (*n* = 35)			p^c^
P-CPQ	M^a^	Md^b^	SD	*M* ^a^	*Md* ^b^	*SD*	M^a^	Md^b^	SD	
Total scale	13.10	10.50	13.15	*26.56*	*21.00*	*17.31*	27.02	23.00	20.99	.000
Subscales										
OS	4.65	4.00	3.65	*6.21*	*6.00*	*3.99*	6.11	6.00	4.49	.084
FL	3.66	2.00	5.09	*7.86*	*7.00*	*5.34*	9.22	8.00	7.72	.000
EWB	3.03	2.00	3.57	*5.69*	*4.00*	*5.98*	5.25	4.00	5.98	.038
SWB	1.75	0.00	3.19	*6.78*	*5.00*	*6.29*	6.42	4.00	7.01	.000

^a^ mean ^b^median ^c^*p*-values obtained from Kruskal-Wallis test

## Discussion

The objective of this study was to evaluate the psychometric properties of the French version of the P-CPQ and the results show that its reliability and validity are acceptable. The process of translation and cross-cultural adaptation was carefully conducted in the line of the criteria described by Guillemin et al. and Beaton et al. [20, 22]. The back-translated version of P-CPQ obtained was very similar to the original and kept its 31 items. Its reliability is acceptable and converges with the Cronbach α values higher than 0.80 obtained by Goursand et al. for the Brazilian version (0.84) and by Albites et al. (0.84). (15.17) However, the internal consistency of the “Oral Symptoms” domain is more moderate. This value is somewhat different to those reported by Jokovic for the English version [9] and MacGrath for the Chinese version [6] but similar to that of Albites for the Peruvian Spanish version [16]. The test-retest reliability revealed good reproducibility with an ICC higher than 0.70 for the total score. The ICC values ranged, from 0.63 to 0.76 for the domains, indicating substantial agreement. These results are satisfactory as values of 0.5 or above are considered acceptable according Cronbach et al. [25]. Similar results were found in the Brazilian and Chinese’ studies [6, 14].

Regarding construct validity, in the present study the rank correlations between the global ratings of oral health and overall wellbeing, and the total scale score and the scores of the domains, were positive and statistically significant. These correlations ranging from (*r* = 0.36 to *r* = 0.54) could be interpreted as being of weak to moderate strength. Although these correlations were weak, it should be noted that this is consistent with previous studies: in the Peruvian Spanish [16] and Brazilian versions [14], and similar results in the validation of the Chinese version of P-CPQ [6].

Factor analysis also examined the construct validity of the proposed factors. The EFA identified seven factors explaining 65,23% of the variances. The four factors retained suggested by the original construct explained 47,24% of the cumulative variance and showed a poor model fit. These findings are in agreement with those reported in the previous study which found that the initial factor analysis of PCPQ 31 items extracted 8 factors with eigenvalues > 1 and the four-factors model did not exhibit a clear factor solution even after rotation [29]. The original P-CPQ instrument [9] proposed 4 subscales: OS, FL, EWB and SWB, but in this study, due to linguistic and cultural differences, the first (OS) and second (FL) factors were split into 3 or 4 factors, respectively. It is assumed that parents perceived oral symptom and functional limitations in different ways.

To discuss the model fit of PCFA, it has been suggested that RMSEA values lower than 0.05 are good. Therefore the RMSEA value of 0.049 in this sample indicate a good fit. The CFI value is higher than 0.90, which shows a relatively good model fit according to HU and Bentler [27, 28]. The fit indices, NFI and TLI should be higher than 0.90 for a good fit but in this sample, the two indices are a little below the criterion. Although the TLI did not reach the cut-off value, it was quite close to 0.90. These two indices are known to be depending of the sample size. Based on these fit indices, this sample has an acceptable fit to the 7-factor model. It might be assumed that these discrepancies could be related to linguistic and cultural specificities. For this, subscales should be reworded to better reflect self-perception of quality of life. To our knowledge, this is the first study reporting a cross-validation of the P-CPQ 31 items using PCFA.

When testing discriminant validity, the French version of the P-CPQ showed it was clearly capable of distinguishing differences of perceptions of impacts as a function of the seriousness of the children’s oral conditions status. But the results could have been influenced by socio-economical status, as it is known that health literacy, for instance, has an impact on the perceived seriousness of disease. A limitation of this study is the sampling method. The data was taken from a convenience sample unlikely to represent the general population. Ideally, a larger sample size would have been better but the number of 142 respondents is similar to the numbers reported by the studies of Jokovic et al. [9], Goursand et al. [12] and Olivieri et al. [18] Generally, a total of < 40% respondents selecting “never” or “always” indicated that an item does not show significant “floor” or “ceiling” effects, respectively [30]. Therefore, the floor effect found in this study would not mean that items responses options were not appropriate. They were due to the high proportion of parents-caregivers of healthy children who chose “never” option.

The P-CPQ contains items for which a “don’t know” response is permitted. According to the authors who developed the questionnaire, the exclusion of “don’t know” responses leads to the loss of valuable data. For this raison, all “don’t know” responses were recorded as zero without affecting the performance of the questionnaire [9]. Furthermore, the treatment of “don’t know” (DK) responses have been largely debated by Marshman et al. and they concluded that replacement of DK responses with zero demonstrated acceptable internal consistency and test retest reliability of the total scale and the exclusion of DK responses had a detrimental effect [17]. This is in accordance with previous study [13].

This cross sectional study did not allow testing the responsiveness of the French-P-CPQ. Therefore an additional study is required to explore this further as in the recent case of a separate study performed by Antunes et al. for the Brazilian version of P-CPQ [15].

## Conclusion

This French P-CPQ version showed reliability and validity comparable to the previous versions. The seven-factor model was supported by the PCFA results. However, the cross-cultural structure of the subscales should be further evaluated. Future research should attempt to confirm the EFA derived model.

## Additional file

Additional file 1: Final French version of the P-CPQ questionnaire. (DOC 95 kb)
