# Supplementary material for: Parental–Caregivers Perceptions Questionnaire (P-CPQ): translation and evaluation of psychometric properties of the French version of the questionnaire
Source: BMC Oral Health. 2018 Dec 11;18:211. doi: 10.1186/s12903-018-0670-8 (PMC6290524; doi:10.1186/s12903-018-0670-8)
Supplement: Supplementary file 1 — Final French version of the P-CPQ questionnaire. (DOC 95 kb) [file 12903_2018_670_MOESM1_ESM.doc]

| Original version | French version |
| --- | --- |
| SECTION 1: Child’s oral health and wellbeing | SECTION 1: *La santé bucco-dentaire et le bien-être de l’enfant.* |
| a. How would you rate the health of your child’s teeth, lips, jaws and mouth?  **☐**Excellent  **☐**Very good  **☐**Good  **☐**Fair  **☐**Poor  b. How much is your child’s overall wellbeing affected by the condition  of his/her teeth, lips, jaws or mouth?  **☐**Not at all  **☐**Very little  **☐**Some  **☐**A lot  **☐**Very much  **SECTION 2: The following questions ask about symptoms and discomfort that children may experience due to the condition of their teeth, lips, mouth and jaws!**  During the last 3 months, how often has your child had:  1. Pain in the teeth, lips, jaws or mouth?  **☐**Never  **☐**Once or twice  **☐**Sometimes  **☐**Often  **☐**Everyday or almost everyday  2. Bleeding gums?  **☐**Never  **☐**Once or twice  **☐**Sometimes  **☐**Often  **☐** Everyday or almost everyday  3. Sores in the mouth?  **☐**Never  **☐**Once or twice  **☐**Sometimes  **☐**Often  **☐** Everyday or almost everyday  4. Bad breath?  **☐**Never  **☐**Once or twice  **☐**Sometimes  **☐**Often  **☐** Everyday or almost everyday  5. Food stuck in the roof of the mouth?  **☐**Never  **☐**Once or twice  **☐**Sometimes  **☐**Often  **☐** Everyday or almost everyday  6. Food caught in or between the teeth?  **☐**Never  **☐**Once or twice  **☐**Sometimes  **☐**Often  **☐** Everyday or almost everyday  7. Difficulty biting or chewing foods such as fresh apple, corn on the cob or  firm meat?  **☐**Never  **☐**Once or twice  **☐**Sometimes  **☐**Often  **☐** Everyday or almost everyday  During the last 3 months, because of his/her teeth, lips, mouth, or jaws,  how often has your child:  8. Breathed through the mouth?  **☐**Never  **☐**Once or twice  **☐**Sometimes  **☐**Often  **☐** Everyday or almost everyday  9. Had trouble sleeping?  **☐**Never  **☐**Once or twice  **☐**Sometimes  **☐**Often  **☐** Everyday or almost everyday  10. Had difficulty saying any words?  **☐**Never  **☐**Once or twice  **☐**Sometimes  **☐**Often  **☐** Everyday or almost everyday  11. Taken longer than others to eat a meal?  **☐**Never  **☐**Once or twice  **☐**Sometimes  **☐**Often  **☐** Everyday or almost everyday  12. Had difficulty drinking or eating hot or cold foods?  **☐**Never  **☐**Once or twice  **☐**Sometimes  **☐**Often  **☐** Everyday or almost everyday  13. Had difficulty eating foods he/she would like to eat?  **☐**Never  **☐**Once or twice  **☐**Sometimes  **☐**Often  **☐** Everyday or almost everyday  *During the last 3 months, because of his/her teeth, lips, mouth, or jaws, how often has your child:*  14. Had diet restricted to certain types of food (e.g. soft food)?  **☐**Never  **☐**Once or twice  **☐**Sometimes  **☐**Often  **☐** Everyday or almost everyday  SECTION 3: *The following questions ask about the effects that the condition of children’s teeth, lips, mouth and jaws may have on their feelings and everyday activities*  *During the last 3 months, because of his/her teeth, lips, mouth or jaws, how often has your child been:*  15. Upset?  **☐**Never  **☐**Once or twice  **☐**Sometimes  **☐**Often  **☐** Everyday or almost everyday  16. Irritable or frustrated?  **☐**Never  **☐**Once or twice  **☐**Sometimes  **☐**Often  **☐** Everyday or almost everyday  17. Anxious or fearful?  **☐**Never  **☐**Once or twice  **☐**Sometimes  **☐**Often  **☐** Everyday or almost everyday  *During the last 3 months, because of his/her teeth, lips, mouth or jaws, how often has your child:*  18. Missed school (e.g. pain, appointments, surgery)?  **☐**Never  **☐**Once or twice  **☐**Sometimes  **☐**Often  **☐** Everyday or almost everyday  19. Had a hard time paying attention in school?  **☐**Never  **☐**Once or twice  **☐**Sometimes  **☐**Often  **☐** Everyday or almost everyday  20. Not wanted to speak or read out loud in class?  **☐**Never  **☐**Once or twice  **☐**Sometimes  **☐**Often  **☐** Everyday or almost everyday  21. Not wanted to talk to other children?  **☐**Never  **☐**Once or twice  **☐**Sometimes  **☐**Often  **☐** Everyday or almost everyday  22.Avoided smiling or laughing when around other children?  **☐**Never  **☐**Once or twice  **☐**Sometimes  **☐**Often  **☐** Everyday or almost everyday  *During the last 3 months, because of his/her teeth, lips, mouth or jaws, how often has your child:*  23. Worried that he/she is not as healthy as other people?  **☐**Never  **☐**Once or twice  **☐**Sometimes  **☐**Often  **☐** Everyday or almost everyday  24. Worried that he/she is different than other people?  **☐**Never  **☐**Once or twice  **☐**Sometimes  **☐**Often  **☐** Everyday or almost everyday  25. Worried that he/she is not as good-looking as other people?  **☐**Never  **☐**Once or twice  **☐**Sometimes  **☐**Often  **☐** Everyday or almost everyday  26. Acted shy or embarrassed?  **☐**Never  **☐**Once or twice  **☐**Sometimes  **☐**Often  **☐** Everyday or almost everyday  27. Been teased or called names by other children?  **☐**Never  **☐**Once or twice  **☐**Sometimes  **☐**Often  **☐** Everyday or almost everyday  28. Been left out by other children?  **☐**Never  **☐**Once or twice  **☐**Sometimes  **☐**Often  **☐** Everyday or almost everyday  29. Not wanted or been unable to spend time with other children?  **☐**Never  **☐**Once or twice  **☐**Sometimes  **☐**Often  **☐** Everyday or almost everyday  30. Not wanted or been unable to participate in activities such as sports,  clubs, drama, music, school trips?  **☐**Never  **☐**Once or twice  **☐**Sometimes  **☐**Often  **☐** Everyday or almost everyday  31. Worried that he/she has fewer friends?  **☐**Never  **☐**Once or twice  **☐**Sometimes  **☐**Often  **☐** Everyday or almost everyday  *During the last 3 months, how often has your child been:*  32. Concerned what other people think about his/her teeth, lips, mouth or jaws?  **☐**Never  **☐**Once or twice  **☐**Sometimes  **☐**Often  **☐** Everyday or almost everyday  33. Asked questions by other children about his/her teeth, lips, mouth or  jaws?  **☐**Never  **☐**Once or twice  **☐**Sometimes  **☐**Often  **☐**Everyday or almost everyday  SECTION 4 : Child’s gender and gender   1. **Your child is:  M**  **☐ Male**   **☐ Female**  **Your child’s age is: ______YEARS**  **Questionnaire completed by:**  **☐** Mother  **☐** Father  **☐** Other  **Date completed:**  ______________  **_______ / _______ / _______**  DA Y MONTH YEAR | a. *Comment évaluez-vous la santé des dents, lèvres, mâchoires ou bouche de votre enfant*  **☐**Excellente  **☐**Très bonne  **☐**Bonne  **☐**Moyenne  **☐**Mauvaise  b. *A quel point le bien-être de votre enfant est affecté par l’état de ses dents, lèvres, bouche ou mâchoires.*  **☐**Pas du tout  **☐**Très peu  **☐** Un petit peu  **☐** Beaucoup  **☐** Enormément  **SECTION 2 : *Les questions suivantes portent sur les symptômes et l’inconfort que les enfants peuvent ressentir à cause de l’état de leurs dents, lèvres, bouche ou mâchoires.***  *Au cours des trois derniers mois,* *combien de fois votre enfant a t-il eu:*  1. Mal aux dents, aux lèvres, aux mâchoires ou à la bouche?  **☐**Jamais  **☐**Une ou deux fois  **☐**Quelques fois  **☐**Souvent  **☐** Tous les jours, ou presque tous les jours  2. Les gencives qui saignent?  **☐**Jamais  **☐**Une ou deux fois  **☐**Quelques fois  **☐**Souvent  **☐** Tous les jours, ou presque tous les jours  3. Des endroits douloureux dans la bouche?  **☐**Jamais  **☐**Une ou deux fois  **☐**Quelques fois  **☐**Souvent  **☐** Tous les jours, ou presque tous les jours  4. Une mauvaise haleine ?  **☐**Jamais  **☐**Une ou deux fois  **☐**Quelques fois  **☐**Souvent  **☐** Tous les jours, ou presque tous les jours  5. De la nourriture collée au palais?  **☐**Jamais  **☐**Une ou deux fois  **☐**Quelques fois  **☐**Souvent  **☐** Tous les jours, ou presque tous les jours  6. De la nourriture coincée dans ou entre les dents?  **☐**Jamais  **☐**Une ou deux fois  **☐**Quelques fois  **☐**Souvent  **☐** Tous les jours, ou presque tous les jours  7. Des difficultés à croquer ou à mâcher de la nourriture comme une pomme ou un steak?  **☐**Jamais  **☐**Une ou deux fois  **☐**Quelques fois  **☐**Souvent  **☐** Tous les jours, ou presque tous les jours    *Au cours des trois derniers mois, à cause de ses dents, lèvres, bouche ou mâchoires, combien de fois votre enfant a-t-il :*  8. Respirer par la bouche?  **☐**Jamais  **☐**Une ou deux fois  **☐**Quelques fois  **☐**Souvent  **☐** Tous les jours, ou presque tous les jours  9. Eu des difficultés à dormir?  **☐**Jamais  **☐**Une ou deux fois  **☐**Quelques fois  **☐**Souvent  **☐** Tous les jours, ou presque tous les jours  10. Eu des difficultés à prononcer des mots quels qu'ils soient ?  **☐**Jamais  **☐**Une ou deux fois  **☐**Quelques fois  **☐**Souvent  **☐** Tous les jours, ou presque tous les jours  11. Pris plus longtemps que les autres pour manger son repas?  **☐**Jamais  **☐**Une ou deux fois  **☐**Quelques fois  **☐**Souvent  **☐** Tous les jours, ou presque tous les jours  12. Eu des difficultés à boire ou à manger de la nourriture chaude ou froide?  **☐**Jamais  **☐**Une ou deux fois  **☐**Quelques fois  **☐**Souvent  **☐** Tous les jours, ou presque tous les jours  13. Eu des difficultés à manger de la nourriture qu’il aurait aimé manger ?  **☐**Jamais  **☐**Une ou deux fois  **☐**Quelques fois  **☐**Souvent  **☐** Tous les jours, ou presque tous les jours  *Au cours des trois derniers mois, à cause de ses dents, lèvres, bouche ou mâchoires, combien de fois votre enfant a-t-il :*  14. Du limiter son alimentation à certains types d’aliments? (ex : aliments mous)  **☐**Jamais  **☐**Une ou deux fois  **☐**Quelques fois  **☐**Souvent  **☐** Tous les jours, ou presque tous les jours  SECTION 3: *Les questions suivantes concernent l’impact de l’état des dents, lèvres, bouches ou mâchoires sur leurs ressentis et leurs activités quotidiennes.*  *Au cours des 3 derniers mois, à cause de ses dents, lèvres, de sa bouche ou de ses mâchoires, combien de fois votre enfant a-t’il été ?*  15. Contrarié?  **☐**Jamais  **☐**Une ou deux fois  **☐**Quelques fois  **☐**Souvent  **☐** Tous les jours, ou presque tous les jours  16. Irritable ou frustré?  **☐**Jamais  **☐**Une ou deux fois  **☐**Quelques fois  **☐**Souvent  **☐** Tous les jours, ou presque tous les jours  17. Anxieux ou craintif ?  **☐**Jamais  **☐**Une ou deux fois  **☐**Quelques fois  **☐**Souvent  **☐** Tous les jours, ou presque tous les jours  *Au cours des 3 derniers mois, à cause de ses dents, lèvres, de sa bouche ou de ses mâchoires, combien de fois votre enfant a-t’il :*  18. Manqué l’école (par exemple à cause des douleurs, d’un rendez-vous, ou d’une intervention)?  **☐**Jamais  **☐**Une ou deux fois  **☐**Quelques fois  **☐**Souvent  **☐** Tous les jours, ou presque tous les jours  19. A eu du mal à être attentif à l’école?  **☐**Jamais  **☐**Une ou deux fois  **☐**Quelques fois  **☐**Souvent  **☐** Tous les jours, ou presque tous les jours  20. N’a pas voulu parler ou lire à haute voix en classe?  **☐**Jamais  **☐**Une ou deux fois  **☐**Quelques fois  **☐**Souvent  **☐** Tous les jours, ou presque tous les jours  21. N’a pas voulu parler avec d’autres enfants?  **☐**Jamais  **☐**Une ou deux fois  **☐**Quelques fois  **☐**Souvent  **☐** Tous les jours, ou presque tous les jours  22. A évité de sourire ou de rire en présence d’autres enfants autour ?  **☐**Jamais  **☐**Une ou deux fois  **☐**Quelques fois  **☐**Souvent  **☐** Tous les jours, ou presque tous les jours  *Au cours des 3 derniers mois, à cause de ses dents, lèvres, de sa bouche ou de ses mâchoires, combien de fois votre enfant a-t’il :*  *23.* été inquiet de ne pas être en aussi bonne santé que les autres?  **☐**Jamais  **☐**Une ou deux fois  **☐**Quelques fois  **☐**Souvent  **☐** Tous les jours, ou presque tous les jours  24. été inquiet d’être différent des autres?  **☐**Jamais  **☐**Une ou deux fois  **☐**Quelques fois  **☐**Souvent  **☐** Tous les jours, ou presque tous les jours  25. été inquiet de ne pas être aussi beau que les autres?  **☐**Jamais  **☐**Une ou deux fois  **☐**Quelques fois  **☐**Souvent  **☐** Tous les jours, ou presque tous les jours  26. A été timide ou gêné?  **☐**Jamais  **☐**Une ou deux fois  **☐**Quelques fois  **☐**Souvent  **☐** Tous les jours, ou presque tous les jours  27. A été embêté ou moqué par d’autres enfants?  **☐**Jamais  **☐**Une ou deux fois  **☐**Quelques fois  **☐**Souvent  **☐** Tous les jours, ou presque tous les jours  28. A été mis à l’écart par d’autres enfants ?  **☐**Jamais  **☐**Une ou deux fois  **☐**Quelques fois  **☐**Souvent  **☐** Tous les jours, ou presque tous les jours  29. N’a pas voulu ou n’a pas pu passer du temps avec d’autres enfants?  **☐**Jamais  **☐**Une ou deux fois  **☐**Quelques fois  **☐**Souvent  **☐** Tous les jours, ou presque tous les jours  30. N’a pas voulu ou n’a pas pu participer à des activités comme du sport, du théâtre, de la musique, des sorties scolaires?  **☐**Jamais  **☐**Une ou deux fois  **☐**Quelques fois  **☐**Souvent  **☐** Tous les jours, ou presque tous les jours  31. Eté inquiet d’avoir moins d’amis que les autres?  **☐**Jamais  **☐**Une ou deux fois  **☐**Quelques fois  **☐**Souvent  **☐** Tous les jours, ou presque tous les jours  *Au cours des 3 derniers mois****,*** *combien de fois votre enfant:*  32. A t-il été interrogé par d’autres enfants au sujet de ses dents, lèvres, bouches ou mâchoires?  **☐**Jamais  **☐**Une ou deux fois  **☐**Quelques fois  **☐**Souvent  **☐** Tous les jours, ou presque tous les jours  33. S'est il soucié de ce que pensent les autres de ses dents, lèvres, bouches ou mâchoires?  **☐**Jamais  **☐**Une ou deux fois  **☐**Quelques fois  **☐**Souvent  **☐**Tous les jours, ou presque tous les jours  SECTION 4 :*Age et sexe de votre enfant.*  Votre enfant est :  **☐**Garçon  **☐**Fille  Votre enfant est âgé de :………….ans  Questionnaire complété par:  **☐**Mère  **☐**Père  **☐**Autre  Date:  ----------------  ...../....../......  Jour/Mois/Année |
